# Development and refinement of a complex intervention within cardiac rehabilitation services: experiences from the CADENCE feasibility study

**DOI:** 10.1186/s40814-017-0123-1

**Published:** 2017-02-13

**Authors:** Rachel Winder, Suzanne H. Richards, John L. Campbell, David A. Richards, Chris Dickens, Manish Gandhi, Christine Wright, Katrina Turner

**Affiliations:** 1 University of Exeter Medical School, Exeter, St Luke’s Campus, Exeter, EX1 2LU UK; 2 Royal Devon and Exeter NHS Foundation Trust, Barrack Road, Exeter, EX2 5DW UK; 3 School of Social and Community Medicine, University of Bristol, Canynge Hall, Whatley Road, Bristol, BS8 2PS UK; 4 The National Institute for Health Research Collaboration for Leadership in Applied Health Research and Care West (NIHR CLAHRC West) at University Hospitals Bristol NHS Foundation Trust, Bristol, UK

**Keywords:** Complex intervention, Qualitative research, Depression, Rehabilitation, Behavioural activation, Care coordination, Enhanced psychological care, Intervention development

## Abstract

**Background:**

Patients who experience a cardiac event are at higher risk of developing depression than the general population. Despite this, cardiac rehabilitation (CR) programmes do not provide a systematic approach to psychological care for depression. The CADENCE study aimed to develop and pilot an enhanced psychological care (EPC) intervention consisting of behavioural activation (BA) and mental health care coordination. Following original research commissioning guidance, the intervention was planned to be embedded in routine care and delivered by CR nurses to patients with depression attending CR. This paper describes how qualitative methods were used to develop, embed and refine the intervention.

**Methods:**

This feasibility study involved three CR teams. Observations were made of CR nurses delivering usual care, of EPC training given to nurses, and of supervision sessions provided to the CR nurses. Four nurses were interviewed shortly after their EPC training, and three were interviewed again 6–7 months later having delivered EPC to patients. All nine patients recruited to receive EPC were interviewed. Analyses of the observation notes and interview transcripts focused on how the intervention could be improved in terms of its acceptability and implementation.

**Results:**

Variations were found between the CR teams regarding patient waiting list times, how CR was delivered, what facilities were available and how many CR sessions were offered to patients. EPC was acceptable to both nurses and patients. However, nurses struggled to provide this additional care within their existing workload and resources, and patients’ disrupted progression through the CR programme affected EPC delivery. Limited time and availability of private space meant nurses also delivered EPC by telephone, which was viewed as a pragmatic solution but less preferable than face-to-face. Nurses indicated that patients struggled with some of the written materials. Findings were used to revise the intervention to become a protocol of care coordination which included guided self-help BA.

**Conclusions:**

Insights gained through conducting interviews and observations enabled us to identify barriers to the implementation of EPC, and to modify the intervention to facilitate its delivery within existing services whilst remaining acceptable to both nurses and patients. The multiple method, iterative approach used was key to the success of this qualitative study.

**Trial registration:**

ISRCTN34701576 Registered 29/05/2014.

**Electronic supplementary material:**

The online version of this article (doi:10.1186/s40814-017-0123-1) contains supplementary material, which is available to authorized users.

## Background

Around 19% patients who experience an acute cardiac event report symptoms of depression prior to starting cardiac rehabilitation (CR) [1]. This compares to 2.6% of the general population with depression [2]. The British Association for Cardiovascular Prevention and Rehabilitation (BACPR) stipulate that one of the core components of CR should be to address psychosocial health, assessing for anxiety, depression and quality of life. In addition, psychological factors that could affect behavioural change [3] i.e. lifestyle changes to enhance cardiac outcomes, should be identified and patients with psychological illness should have access to trained psychological practitioners [3].

CR programmes usually involve an initial assessment followed by a structured programme lasting six to eight weeks with up to two contacts per week. A CR session could consist of a clinic appointment where patients were assessed and their cardiac symptoms monitored and discussed, a supervised rehabilitation fitness session, and/or a group educational talk, which may include a talk on stress and relaxation. CR nurses would normally assess for depressive and anxiety symptoms, provide practical advice and reassurance, and refer patients on to other services where available. However, psychological treatment for depressive symptoms is often not available within CR services [1].

CR is offered to patients with an acute coronary syndrome (i.e. myocardial infarction or unstable angina [4]) who have received medical treatment or surgical interventions, such as coronary revascularisation, valve replacement or implantable cardioverter defibrillator. Some patients with heart failure are also offered CR.

The CADENCE Study (NIHR HTA: 12/189/06) [5] was funded to develop and pilot an enhanced psychological care (EPC) intervention for CR patients with symptoms of depression. EPC is a complex intervention consisting of nurse-led-behavioural activation (BA), and mental health care coordination. BA is a therapy that aims to relieve depression by changing people’s behaviour through helping them to see a link between their behaviour and their mood [6]). Patients meeting the study criteria for depression were offered EPC. It was developed by members of the research team (DAR and CD) who are experts in BA and care coordination, and who had previously used and tailored both components for other clinical trials [7–9]. EPC was to be embedded in routine CR care [10], and delivered to individual patients on a face-to-face basis by CR nurses, within their current workloads and within existing CR care pathways.

The process of intervention development is an important element in the implementation of complex interventions [11]. The success of a trial will depend on the intervention being theoretically grounded and delivered as intended, as well as practitioners and participants being willing to implement it [12]. Involving key stakeholders and PPI contributors early on in this process can provide useful insights [13, 14], and qualitative methods can highlight service variation, detect potential barriers to integrating a new service and indicate whether the intervention can be delivered as envisaged [12]. Qualitative methods are viewed as particularly helpful at this early stage, as they can respond flexibly to issues as they emerge [14] and encourage an iterative approach to intervention development [11, 15, 16].

Despite the importance that has been placed on intervention development, and the role of qualitative methods within it, having reviewed the literature we only identified four published examples of how researchers have used qualitative methods to refine and embed a health care intervention for low mood in clinical practice [13, 17–19]. Many studies have described qualitative methods at earlier stages but do not provide details on how the findings were used to refine the intervention once it has been embedded in practice [20–22].

Consistent with the Medical Research Council framework [12], the CADENCE Study included a two-phase evaluation: a feasibility study and external pilot cluster randomized controlled trial. Feasibility studies are conducted in preparation for a main study and assess whether it would be feasible to undertake the main study and if so, how this should be done [23]. One aim of the CADENCE feasibility study was to determine whether it would be possible to develop, refine and embed EPC within existing CR services in a way that would be acceptable to both patients and CR nurses. This paper describes how qualitative research methods were used to achieve this.

## Methods

### CADENCE feasibility study

#### The Intervention

Evidence indicates that BA is as effective as cognitive behavioural therapy in the management of depression [24–27] but simpler for non-specialists to deliver [9] and less intense for patients to implement. The mental health care coordination element of the intervention was based on current UK National Institute for Health and Clinical Excellence (NICE) guidance [28]. It entailed regular review of symptoms, involving participants in decisions about their treatment, and referral to their GP or existing community/primary care mental health services if necessary, either during or on exiting EPC. Coordinated care can enhance outcomes for depression [8], especially when a psychological intervention for depression is included [29]. Nurse-led depression management has been shown to be an effective treatment across a range of long term health conditions [10]. The intervention was to include a form of BA of moderate intensity (i.e. some elements of higher intensity BA, such as functional analysis of the connection between mood and behaviour, rather than simply listing valued activities to add back into their lives), and to nurture a mental health care coordination role for CR nurses.

#### Early patient involvement

Early on in the study, the intervention was discussed with the research team’s Patient and Public Involvement (PPI) group. This group was formed at the start of the study. It included four individuals who had experience of cardiac events (either personally or via a relative), of BA and/or depression. Throughout the study’s duration, each individual was invited to team meetings and to comment on the study materials, procedures and dissemination of findings, in the context of their own experiences.

#### Recruitment, training and supervision of nurses

Three CR teams based in the South West of England were recruited. Four nurses within the three teams were trained to deliver EPC [30]. Nurses were trained together over two days by DAR and CD. The training covered: BA and its delivery, mental health care coordination, assessment and management of psychiatric risk, and how to use the CADENCE materials. During training nurses had opportunities to practice EPC skills and to discuss any other concerns they had about delivering EPC in practice. They were also given a manual, which had been developed by DAR and CW (Richards DAR: Cadence nurse handbook version 5, unpublished). The manual covered these areas, and included a BA session delivery guide, and hard copies of clinical materials to use during BA sessions.

Whilst delivering the intervention, nurses also received supervision from a clinical supervisor (CD or PM). Supervision was held by telephone on a weekly basis with nurses one at a time. It gave nurses an opportunity to discuss with a supervisor their experiences of delivering EPC and any difficulties that had arisen. Supervisors, in turn, offered guidance and support. Supervisors wrote notes about each supervision session and provided the research team a written report once nurses had stopped delivering EPC.

#### Recruitment of patients

The trained CR nurses screened all patients at their initial CR assessment and offered the intervention to participants identified as having depression (score of ≥ 10 on the Patient Health Questionnaire-9 (PHQ-9) [31, 32]). Using the PHQ-9 meant a change from their pre-existing assessment procedure, which was the Hospital Anxiety and Depression Scale (HADS) [33]. Nurses were asked to use the PHQ-9 because it is highly correlated with diagnostic depression criteria [32] (a study eligibility criterion), and because it is routinely used in UK general practice for the management of depression, and thus its adoption would aid the care coordination element of the EPC intervention.

Patients reaching study criteria were referred on to the research team by the CR nurse. All recruited patients were offered the intervention and were invited to take part in the qualitative study.

#### Delivery of EPC

At the start of the feasibility study it was proposed that nurses would deliver EPC to participating patients once a week, on an individual basis, during any of their CR sessions. Patients recruited to the study were to receive individual treatment programmes of BA and care co-ordination as appropriate, guided by the use of a CADENCE-developed patient handbook developed by DAR (Richards DAR: Cadence participant handbook, version 5.0, unpublished). The patient handbook aimed to support the information provided by nurses on BA and care coordination, and offered guidance and further information on other psychological help available, e.g. local community mental health services.

The EPC sessions were to include symptom monitoring of depression and anxiety using the PHQ-9 and the Generalized Anxiety Disorder-7 (GAD-7) [34] respectively; a review of any risk issues and of the patients’ completed self-monitoring materials, e.g. mood and activity diaries and valued activities sheet; and a functional analysis entailing nurses and patients developing a shared understanding of behaviour patterns linked to normal and low mood and of triggers to ‘depressed behaviours’. In addition, patients were to work with the nurse to identify alternative valued activities (routine, pleasurable and necessary) and schedule in these activities to replace those associated with low mood. Nurses were to care co-ordinate as necessary during these sessions. The initial EPC session was estimated to take between 20 and 30 minutes to give time to discuss BA concepts. Follow-up sessions were expected to be shorter, around 10-20 minutes, according to individual patient needs. Patients could continue to receive EPC up to the time they were discharged from their CR.

### The qualitative component of the feasibility study

The qualitative component of the study employed a range of methods. Data from each method brought different insights and perspectives, and each data set was used to develop and refine the intervention to ensure it was acceptable to both patients and nurses, and feasible to deliver within cardiac rehabilitation services.

#### Site observations

Early on in the study, following discussions with stakeholders (i.e. cardiologists and CR nurses) who indicated CR teams varied in how they delivered CR, a decision was made to conduct site observations. These were unstructured, informal observations, undertaken by RW, to gain some sense of who was delivering CR, where and to whom, and thus to determine how best to embed EPC in clinical practice.

RW aimed to observe each nurse in a range of settings (e.g. public gym, clinic room, physiotherapy room, a gym changing room and a health centre café) within a variety of venues (e.g. hospital, leisure centre, community hospital or health centre) where nurses delivered components of CR. Seven observations of CR activities were conducted across five venues. Activities observed included fitness classes, individual fitness checks and training, hospital or community assessments or review appointments, and a health education presentation. The observations were conducted from August to October 2014, prior to the EPC intervention being implemented by nurses. They were recorded through RW making detailed notes whilst observing. On average, the observations each lasted 87 minutes.

#### Observations and interviews with nurses

During August 2014, both days of nurse training were observed and notes made of any issues they raised. Within four weeks of training, in September 2014, the four nurses were interviewed prior to them commencing EPC delivery. The interviews were semi-structured in nature and conducted by telephone. Their purpose was to explore their views of the training and to identify any problems they anticipated in terms of delivering the intervention in practice. The data gathered would be used to refine the intervention at this early stage if required. Three of the nurses, from the three teams, went on to provide EPC and were interviewed a second time between February and May 2015 once they had delivered EPC to CR patients. These second interviews assessed nurses’ views on delivering EPC in practice, including exploring how nurses varied in how they had delivered EPC, and the extent to which they felt it could be embedded within CR services. They were held in person, at the nurse’s place of work.

Nurses’ delivery of the intervention was also assessed via RW listening in through a speaker phone to two supervision sessions (October to November 2014) and talking to the supervisors immediately after these sessions. In addition, supervisors provided a written report detailing their own observations on nurse progress and what support they had provided over the 25 supervision sessions.

#### Patient interviews

All nine patients recruited to the study were invited to take part in a semi-structured interview to explore their experiences of receiving the intervention, in order to identify ways to refine the intervention to improve patient acceptability. All nine patients agreed to be interviewed. The patient interviews were conducted between February and May 2015. They were held in person, in the patients’ own home.

During both the nurse and patient interviews, topic guides were used to ensure consistency across the interviews. The main areas covered in the topic guides are listed in an additional file [see Additional file 1]. The content of the topic guides was informed by the aims of the interview, relevant literature, the researcher’s knowledge of EPC, and insights gained during the earlier observations and discussions. With participant consent, the interviews were audio-recorded and transcribed verbatim. RW collected all the qualitative data.

### Analyses

Insights gained through the observations conducted at CR sites and during nurse training needed to be quickly fed back to the research team, so that changes to the intervention could be made prior to the nurses conducting EPC. Thus, analysis of the notes taken during the observations focused solely on identifying information relevant to the design and delivery of the intervention. RW read and re-read her field notes, and then highlighted relevant findings to the rest of the team.

Analysis of the nurse and patient interview data was thematic, and focused on how the intervention could be improved in terms of its acceptability and on the extent to which it could be effectively delivered in practice. Six transcripts from nurse and participant interviews were read and re-read by two members of the research team (KT and RW) to gain an overview of the accounts given, identify emerging themes and to develop preliminary coding frames for each interview data set. RW purposefully sampled these transcripts on the basis that the content of the interviews had been very different. KT and RW independently coded the transcripts and then met to discuss their interpretation of the data and preliminary coding. The coding frames were then revised, with new codes developed and existing codes defined more clearly or deleted. The coding frames were developed alongside each other to ensure they included similar codes where common areas had been explored with both patients and nurses, or where common themes had emerged from the data. For example, both nurse and patient coding frames included the code for experiences of EPC delivered face-to-face versus by telephone. Doing this meant data pertaining to these codes could be analysed and the accounts of patients and practitioners triangulated.

An approach based on Framework analysis [35] was then used to summarise the data. This entailed summarising the data in a table where each row represented a participant and each column heading was based on the codes that had been developed, rather than pre-defined headings, which is usually the case when using Framework analysis. Doing this enabled comparisons to be made within and across the data. Data from the nurse interviews were analysed separately from those gathered from patients, before accounts from nurses and patients were triangulated.

**Fig. 1:**
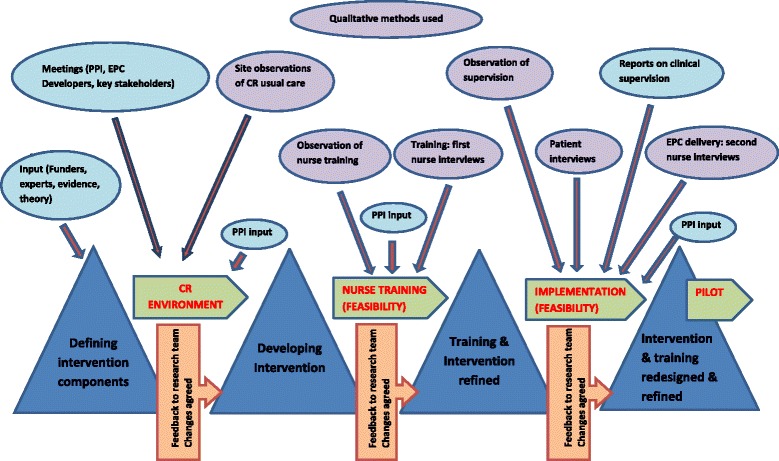
Sequence of methods to inform stages of intervention development

Findings from the observations and interviews were recorded on a table and reported to the intervention development team (DAR, CD, SR, JC and CW) and wider research team at three time points during the feasibility study (Fig. 1), so that recommendations for changes could be discussed and amendments made to the intervention at key stages.

Quotes have been reproduced below. These quotes were selected for their relevance to the topic and their ability to illustrate the points made. They are tagged according to type of interviewee (nurse or patient), individual patient or nurse identifier number and, where more than one interview was conducted, whether it was a first or second interview.

## Results

### CR nurse teams and participant characteristics

Table 1 describes the characteristics of patient and nurse interview groups. All nine patients reported initially having sudden chest pain and/or felt acutely unwell and were taken to hospital. Eight patients said they had had a heart attack and the ninth was unsure of his diagnosis. Seven patients said they received one or more surgical interventions and two were treated with medication alone. Three of the nurses from three different teams went on to deliver EPC to patients. Six of the nine patients said they had received between 3-12 BA sessions. One patient had dropped out after three sessions because he felt he could not cope with completing the diary and was not benefitting from BA. Three patients said they had received no CR, one saying she was waiting for their condition to stabilise before starting CR and one because his CR had been postponed due to a recent bereavement and the third, had preferred to manage his rehabilitation himself at home. Two of the three patients had said in the meantime that their scores had improved.

**Table 1:** Characteristics of patient and nurse interview groups at time of interview

	Patients (N = 9)	Nurses (first interview) (N = 4)	Nurses (second interview) (N = 3)
Gender: male/female	7/2	1/3	1/2
Age (mean years)	60.6	Not asked	Not asked
Ethnicity - white	9	4	3
Mean length of interview (minutes)	44.6	29	56
Type of cardiac event reported^a^			
Heart attack	8		
Coronary Artery bypass graft	3		
Insertion of stent(s)	5		
Valve surgery	1		
Returned to hospital (complications/concerns)	6		
EPC sessions received/provided (range)	0-12	N/A	3–12

^a^Some patients had experienced more than one cardiac event

Table 2 describes the characteristics of the CR nurse teams. Two of the three teams consisted of one CR nurse working within the community (each covering separate areas). The third team was hospital-based and included CR nurses who also worked within the cardiac unit. Each team provided one or more CR fitness programmes. The hospital-based nurses offering a rolling programme, i.e. patients could start the programme at any time point, and education talks. The community based nurses provided programmes that started on scheduled dates within a number of community settings and which ran over six to eight weeks.

**Table 2:** Characteristics of CR nurse teams participating in the feasibility study

	Team I	Team II	Team III
Size of nurse team	4 or more^a^	1	1
Participating CR nurses	2	1	1
Where based	Hospital	Community	Community
Number of sites used for CR	2	6	6
Number of CR sessions (including fitness programme) usually on offer	12	Up to 12	Up to 12
Timescale of fitness programme	6	8–12 weeks	8–12 weeks

^a^Nurses from the cardiac ward sometimes helped with the CR programmes

Having analysed the various data sets, four main headings were identified under which findings from discussions, observations, supervisory notes and interviews could be presented. Below, findings are detailed under these headings.

### Variability of CR team organisation and supporting infrastructure

Discussions with stakeholders, along with the site and training observations, highlighted wide variations between the CR teams recruited to the study in terms of team type (hospital or community based), size (single nurses versus larger hospital team), methods of rehabilitation delivery (one-to-one clinic, ongoing or set dates for fitness programmes, home DVD), and number of sessions and timescale over which they were offered (six or 12 sessions delivered over eight to 12 weeks). There was also variability in the use of session time (clinic or fitness centre depending on clinic and venue availability), type and amount of private space available (e.g. clinic room versus space in the corner of the gym changing room), waiting list times (0-6 weeks) and local facilities on offer (e.g. health centre with gym and café attached or room within a charity building). Payments incurred whilst attending a session varied too, e.g. those receiving CR in the community were required to pay for each fitness class, whereas those having CR in the hospital paid for their parking only.

Key concerns voiced by nurses relating to the team organisation and infrastructure at this early stage were the lack of a private space to deliver EPC, nurses needing to work alone which would make it challenging to focus on one patient during or after a fitness session, and fitting in the extra time required to deliver the intervention.

### Nurses and patients’ views on incorporating EPC into CR services

Both nurses and patients commented that they thought it appropriate and timely to provide an intervention within CR that aimed to support patients’ mental wellbeing. Patients described how their cardiac event had left them feeling *‘quite naked …, vulnerable’* (patient 1), *frightened* (patient 6, patient 4, patient 7), ‘*scared*’ (patient 5) or alone, and *’very, very low’* (patient 7):

*‘Well the first about 10 or 14 days I was proper down and was thinking “what, why did I have it done”? And “why is it happened to me” you know and I just sat there, I had an armchair there then and I just sat there looking at the telly and thinking “oh god”.'* (patient 3).

Patients described how they were keen to have the opportunity to discuss such feelings with a CR nurse.

However, one male patient commented that he felt uncomfortable discussing emotional issues with a female nurse:

*'Personally, I prefer the lady who was with me* [the CR nurse]*, to just concentrate on that, whether my heart gets faster or not on that machine.'* (patient 1).

Furthermore, several patients commented that more peer support would be helpful, and one individual stated that a group setting for BA delivery would be *‘one hundred times more effective’* (patient 4). Like others, this patient felt that the opportunity to talk with others who had been through a similar situation was lacking, and he felt that providing BA in a more relaxed group environment to all CR patients (low mood or otherwise) would *‘normalise’* the sessions (patient 4).

Nurses remarked how providing EPC had given them new ways of supporting their patients and the training had provided them with a pathway to deal more fully with patients’ mental health issues. In terms of mental health care coordination, one nurse commented that they had always seen coordinating patient care as part of their CR role: in the past, the nurse had made referrals to the patient’s GP and on occasion, to a psychologist. However, BA was new to all nurses and three had not been aware of other existing specialist community mental health services or referral options prior to the training:

‘*I didn’t know anything about mental healthcare co-ordination at all before, not at all. I wouldn’t have known where to go and I wouldn’t have known what to do so it opened up a lot of avenues for us definitely.’* (nurse 1, first interview).

Patients said they were not aware of the care coordination process or any referral to other services during EPC, but had occasionally been asked by the nurse if they would like a referral elsewhere. At the time, patients had not felt this was necessary. Nurses had also advised patients to see their GP. Patients usually viewed this as acceptable:

*‘She* [the CR nurse] *told me to go and see my doctor, she said she would refer me to my doctor which I did.’* (patient 7).

### Nurses’ views and experiences of delivering the intervention

Nurses were very positive about moving from using the HADS to using the PHQ-9 and GAD-7 to screen and monitor patients for depression and anxiety. They described how their CADENCE training and the new measures gave them confidence to discuss risk issues that arose, e.g. thoughts about suicide or self-harm that were identified when using the PHQ-9, but not using the HADS. Their increasing confidence in discussing risk was also due to the fact that the research team had provided them with a clear pathway to follow if a patient was identified as being ‘at risk’.

*‘The HAD score was not that helpful and didn’t give an indication of risk … using GAD-7 and PHQ-9 instead of HAD, means that the screening is more efficient. I think we are much more aware of risk management and risk and what to do.’* (nurse 3, second interview).

It was clear from observing supervision sessions, from the supervisor’s report, and from nurse and patient interviews, that a number of factors had prevented patients from regularly attending EPC sessions within their CR. These included patients experiencing cardiac complications, hospitalisations, unrelated illness and a family bereavement. Nurses found this disjointed flow challenging for EPC delivery:

*‘It’s* [i.e. providing a mental health treatment] *been challenging, extremely challenging, I would say, because nothing is ever straightforward with patients that we’re dealing with. So you think you’re on track with dealing with maybe their mood and then suddenly they’ll have a cardiac event or something else in their life, some other illness and then of course their mood then, isn’t the priority, it’s their physical health, cos obviously with hearts, that’s going to always take priority.’* (nurse 4, second interview).

Cancelled or incomplete EPC sessions regularly needed to be rescheduled. Nurses felt delivering EPC had led to some patients being seen more often than would have occurred in the past:

*‘Because of the BA, I was making sure I was speaking to them next week and then they’d want to come into clinic and it would create more* [clinic contact]*, whereas before we might well have said, “Well, you’ve got physical symptoms, you’re seeing your GP”, or, “You’re seeing the cardiologist in three weeks’ time, give me a ring once you’ve seen them, let me know how you got on and then we’ll make a plan from there”… whereas with BA of course, in that three weeks you could have seen them three times in clinic. … Taking up three half hour slots, so I think that’s probably the reason why it took up more* [time].*’* (nurse 4, second interview).

Two nurses said that they had used their nurse manuals regularly to guide them through the sessions with patients and felt the manuals had worked well. Nurses also suggested more structured sheets for recording and guiding patients through EPC sessions, as nurses recorded details of sessions in different ways, which meant recording was unsystematic and varied from nurse to nurse.

Nurses had found it difficult to persuade patients to read the CADENCE handbook. Patients had described the handbook as too long, and nurses felt some patients were too busy to read it. However, nurses suggested that most patients had engaged in the BA ‘homework’ and this was confirmed in the patient interviews. Homework usually involved completing a weekly mood and activity diary, and identifying valued activities as potential alternatives to activities associated with depression. However, nurses were unsure when to use one particular worksheet intended to aid functional analysis (‘Triggers, Response, Avoidance Pattern’/‘Triggers, Response, Alternative Coping’ (or ‘TRAP/TRAC’)).

Nurses reverted to delivering EPC by telephone, rather than face-to-face, when there was a lack of privacy or time. Even finding privacy to make the call was sometimes a challenge:

*‘I had to sneak up and hide in a room that luckily wasn’t booked, try and find reception to be able to make the call* [for an EPC session], *because I couldn’t make the call in the gym off the telephone as there was people throwing weights around.’* (nurse 3, second interview).

However, nurses felt that reviewing homework was simpler and patients engaged more fully when this was done in person rather than by telephone.

While EPC was seen as potentially beneficial, nurses could not envisage integrating the treatment into their CR sessions, in the long term, in its current form. They found providing the intervention an extra pressure and a source of anxiety:

*‘Sometimes we felt slightly relieved when the PHQ come back, it’s still low* [i.e. not low in mood]*, you’d [sigh], we haven’t got to* [deliver EPC to this patient] *… it was relief when they scored low.’* (nurse 2, second interview).

Nurses commented that patients receiving the intervention had required more contact time (i.e. clinic time, more sessions and more overall contact) than non-CADENCE patients, since the intervention rarely fitted entirely into their CR programme. The EPC course went on for longer or started or ended before patients commenced their CR fitness programme (which was part of their usual care), although in at least one case, and noted by both the nurse and the patient, EPC had encouraged the patient to take up the fitness programme.

While two nurses had only delivered EPC to one patient each, they imagined it would be too challenging to have more than one patient receiving the intervention on their caseloads at one time, due to the impact on their time, clinic space and workloads.

One nurse, who had delivered EPC to four patients, three of whom had received EPC concurrently, commented that the squeeze on her time was the biggest challenge:

*‘I think the* [extra] *time* [required] *effectively, and it has been really difficult, really difficult to do, and I mean I don’t feel like I’ve had a huge number of patients on it, … over the past few months, it shouldn’t have been, but it did have, bearing in mind I’m only a part-time worker, we’re lone workers, that does have an impact on it, … it’s definitely just the time.’* (nurse 4, second interview).

More time, nurse backup and lower workload were seen as important if the intervention was to be delivered. These issues had implications for the nature of EPC delivery in terms of the intensity of input from the nurse (e.g. number and length of EPC sessions).

Two main issues were identified from the supervision observations: a need to more clearly record the detail of EPC sessions within clinical records in order to support clinical supervision, and the need to more clearly define when patients should be discharged from EPC.

### Patients’ views and experiences of receiving the intervention

Some patients admitted to not reading their CADENCE handbook. Reasons given included being dyslexic, too busy or just feeling that their CR nurse would provide them with all they needed to know about EPC. Often when asked about the handbook, patients remembered little about its contents, except for the descriptions of patient scenarios, which some patients identified well. Nevertheless, most patients did complete the weekly mood diaries, although some said they completed or changed the entry retrospectively.

Most patients who engaged with BA during the study said they had developed an awareness of how their behaviour affected their mood, and vice versa. They had benefitted from this by actively changing their behaviour in response to realising they felt low:

*‘When I’m noticing that I’m on a downward spiral, I need to pick up the phone and I need to say, “Can we go out, can we go and have a cup of coffee somewhere, can we go and do something?” Because actually, it breaks the mood. And I think I’ve done that relatively successfully*.’ (patient 4).

Other benefits of BA mentioned by patients were having the opportunity to talk with the nurse about how they were feeling and gaining insight into what triggered low or improving mood. Some patients commented on being more proactive in helping themselves or seeking out professional help (for example from therapists or their GP) to avoid their mood deteriorating too far, and identifying areas in their lives that they needed to address. Several patients commented that the BA element of the intervention specifically, had helped to improve their mood:

*‘So what was great about the CADENCE pilot that I’ve done is it’s shown me that my life/work balance isn’t very good. I probably was aware of it anyway, but it certainly flagged it up. I’ve been trying to walk more and get more exercise generally, and I think by actually noting my mood during the day and during every two hours and jotting it down I learnt quite a bit about myself, which I wasn’t expecting.’* (patient 9).

*‘She told me to keep a diary of what I did throughout the day and that’s, and like what my mood was like, and always just and stuff, basically to work out what was making me put in a bad mood and whatever. And that kind of helped.’* (patient 5).

Two patients, however, felt they had not benefitted from receiving EPC. One was an 85 year old man, who withdrew from the BA component of the intervention as he was finding it difficult to complete the paperwork and was put off by constant mood monitoring:

*‘That’s what was required on a daily and then virtually an hourly* [basis]. *The bit that thoroughly put me off was the fact that I was expected to concentrate on that and know when I was having a low mood. Well, I find these moods suddenly come on you and you don’t think about them, you just try and think about something else.* [Coughs]*. And you have to concentrate on noting when this mood changes. While I’m doing that, I’m not relaxing, I’m thinking about this thing that I’m supposed to be doing and it didn’t make me feel at all comfortable, I mean, it was a question of, “Oh, did I just have a mood change?.”* ‘(patient 2).

The other patient felt that physical and mental aspects of care should be kept separate and he would have preferred to see a male therapist dedicated to providing mental health support.

Patients also felt that the space available for EPC delivery was not always ideal. One man had received EPC sessions in the changing room after the fitness session and in a café bar adjoining the gym. Although he had no issue with this, he recognised that others might feel uncomfortable discussing emotional matters in such public environments. While occasional telephone EPC sessions were seen as acceptable to patients, most preferred face-to-face contact for the sessions.

## Discussion

Through using qualitative methods, we were able to identify the practical challenges of embedding an intervention within existing services, and to consider how this intervention could be developed and refined to optimise its acceptability to both patients and nurses.

The findings suggest embedding a standardised form of EPC in CR services is challenging. CR settings are diverse and care pathways are complicated by patients’ life circumstances and by breaks in therapy due to social and physical problems. While BA is acceptable to both patients and practitioners, and mental health care coordination seemed well suited to the setting, it was clear that the intervention needed to be pragmatic and versatile so that it could adapt to local circumstances and to the specific venue in which it was being delivered [12]. The context and time constraints within which CR nurses work, meant EPC would need to be revised if it was to be routinely delivered within CR programmes long term. The findings implied that we would need to make the nurses’ input less intense and more systematic, and increase the focus on self-help and care coordination (Table 3).

**Table 3:** Description of the Cadence EPC intervention during the feasibility study and changes made for the pilot study (agreed changes in italics)

Feasibility	Pilot study
Nurse-led behavioural activation (BA) sessions supported by patient handbook. Care coordination on exiting BA sessions	*Nurse provision of care coordination with embedded nurse-supported self-help behavioural activation sessions using patient handbook as core material*
Two-day nurse training:	
• Two days, delivered by the intervention developers:	• Two days, delivered by the intervention developers *(or for nurses unable to attend these session, or one day + web-based/DVD training):*
• Assessing and managing risk	• No change
• Explaining BA to patients	• Explaining BA to patients*– more detail*
• Care coordination	• Care coordination – *more detail/practice*
• Behavioural activation, role play and skills practice	• Behavioural activation role play and skills practice
• Ending the Cadence Programme	• Ending the Cadence Programme *– more detailed information*
• Support available	• Support available *(more information on optimising supervision provision)*
Nurse manual and other materials for EPC delivery:	
• Short guide to delivering BA and separate care coordination manual	• *Longer guide, incorporating care coordination and BA, with more structured session guides for BA and guidance throughout*
	• *Written memory aids for nurses to use in discussion with patients to cover: introducing EPC concepts, explaining treatment options, regular review and BA.*
Patient Handbook	
• BA handbook for participants to take home, read and follow:	
o Plain cover, very similar to the nurse manual.	o *Shorter* BA handbook for participants to take home, read and follow: *Colour coded to be distinct from nurse manual*
o Patient case studies with a cardiac event and depression	o Patient case studies with a cardiac event *and how their depression resolved*
o Five-step guide to self-guided BA	o No change
o Appendices with examples of the other materials	o No change
Initial nurse/participant EPC appointment:	
• Screen for depressive symptoms using PHQ-9 and GAD-7	• No change
• Discuss nurse-led BA programme	• No change
• Provide mental health care coordination (MHCC) if patient not interested in receiving BA	• *Nurse/participant agree a treatment plan (i.e. BA or referral to specialist services or GP)*
Nurse supervision sessions (by telephone):	
• Weekly individual supervision with experienced clinicians	• *Fortnightly individual or group nurse supervision with an experienced clinician*
• No aids for nurse preparation of supervision sessions	• *Standardised record sheets for nurse preparation of supervision sessions*
Nurse-led BA sessions comprise:	
• Providing the patient with the Cadence BA handbook to use and asking the patient to read through it	• Providing the patient with the Cadence EPC handbook to use; *emphasising the importance of working through and being guided by the handbook*
• Introducing mood/behaviour activity diary for patient to complete/review each week	• No change
• Introducing other materials where appropriate (e.g. *valued activities sheet*, *TRAP/TRAC*) over time	• Introducing other materials where over time. *TRAP/TRAC not included in these materials but other materials added*
• Nurse will guide participant through the programme and patient is given tasks or ‘homework’	• *Nurse will check participants’ understanding and progress through the programme.*
	• *Nurse and patient follow a structured BA session guide*
Mental health care coordination (MHCC):	
• Provision of mental health care coordination if patient prefers not to receive BA or at end of CR programme	• *Regular review of patient’s mental health status (whether or not receiving BA) and care coordination to include onward referral to other services at any point during or at completion of BA as appropriate.*
• Consider referral to existing community/primary care mental health services (e.g. GP, Improving Access to Psychological Therapies (IAPT), CR team psychologist)	• *More formal review of PHQ-9 and overall progress at Session 4. Make decision whether to continue with the current care coordination plan or move to a different approach.*
BA sessions (face-to-face or by telephone):	
Nurse-led delivery of BA by CR nurses during their usual CR sessions:	*Nurse-supported delivery of self-help BA during their usual CR sessions (symptom assessment, risk assessment, review of progress and forward planning):*
• Opportunistic accommodation for BA session e.g. gym changing room	• No change but nurses are asked to consider optimal choices for delivering EPC in terms of space and privacy during their training
• Monitor patient’s mental health using PHQ-9/GAD-7	• No change
• No standardised paperwork for recording number and contents of BA sessions with patients	• *Standardised paperwork for recording number and contents of sessions with patients, to store with patient’s other nursing notes*
• Review PHQ-9/GAD-7 each week if nurse has concerns. BA discontinued if PHQ-9 score drops to < 10 (i.e. depression symptoms improved)	• *Review PHQ-9/GAD-7 regularly, especially if nurse records deterioration or no improvement in mental health.* BA discontinued if PHQ-9 score drops to < 10 (i.e. depression symptoms improved*)*
• BA can continue until patient is discharged from their CR	• *Maximum number of EPC sessions with the nurse = 8*
• Discharge from EPC and provide care coordination at the end of contact with rehabilitation nurse	• Discharge from EPC and provide care coordination *when PHQ-9 score < 10 and/or when up to 8 sessions completed*
• Nurse writes to GP at end of CR programme. At this point, also consider referral to existing community/primary care mental health services (e.g. GP, IAPT, CR team psychologist)	• *Standardised discharge letter back to GP at CR programme end. Consider referral to community/primary care mental health services (e.g. GP, IAPT, CR team psychologist) at any point during the sessions, but especially if no improvement or there is deterioration*

While the nurse training needed to maintain its focus on screening for and managing risk issues, and understanding and explaining BA concepts, it would also need to provide clearer pathways on how each EPC session should be managed and recorded, and how to optimise the use of supervision sessions to support patients. Before moving into the external pilot cluster randomized controlled trial, which was the second component of the CADENCE study, in light of our findings, a number of more major changes were made. Our delivery of BA was revised to be less intense to reduce the logistical/practical challenges faced by nurses delivering care. Greater emphasis is now placed on providing opportunities for mental health care coordination throughout the intervention to cope with patients using the service in non-standard ways. The training now includes asking nurses to consider optimal choices for EPC delivery in terms of space and privacy. We have also listened to nurses and patients and made improvements to their accompanying guidance materials (Richards DAR: Cadence nurse handbook version 5, unpublished and Richards DAR: Cadence participant handbook, version 5.0, unpublished), nurses’ note keeping tools and by making guidelines and recording sheets for care coordination and supervision records clearer, and more systematic. The number of changes we made to the intervention, nurse training and study materials between the CADENCE feasibility study and the pilot trial, highlights the importance of conducting a feasibility study when implementing interventions in real life settings.

### Strengths

Our study benefitted from a flexible, iterative approach, with methods of data collection being chosen in response to new knowledge and with earlier forms of data collection informing what we then focused on during the nurse and patient interviews [12]. This order of methods, i.e. observations and then interviews, also meant we gained insights that enabled us to appreciate and contextualise interviewees’ comments. Using multiple methods was also beneficial because the observational data emphasised the practical challenges about how the intervention could be delivered and within what context, while interview data enabled us to assess what practitioners and patients thought about the intervention and enabled them to raise issues that were salient to them and which we might not have considered. Having multiple data sets enabled us to triangulate findings, which increased the confidence with which we could draw conclusions [15, 16]. Gathering data from both nurses and patients also meant the intervention could be revised in terms of meeting the needs of both parties [14].

### Limitations

The sample of nurses and patients interviewed was small and limited to the South West of England, and all the patients were of a white British ethnic background. While the main themes were evident across the different data sets, the number of observations and interviews we could conduct was limited by the number of teams, patients and nurses recruited to the study. This meant we were unable to continue data collection until data saturation had been reached. Thus, we cannot be confident that further themes would not have emerged if we had observed more sites and held more interviews [36]. These issues limit the extent to which our findings can be generalised to other CR services. Despite this, we gained important insights that otherwise would only have been encountered at the pilot stage or later, and thus at a stage when it would have been problematic to change the intervention. What we do not know, however, is whether the changes that were made to the intervention, in light of our findings, have affected the effectiveness of EPC. The focus here was on improving its acceptability, which means the intervention is more likely to be delivered and received, but may not mean the intervention is more effective in terms of improving symptoms of depression. This may have been the case here, as our findings suggested that patients benefited from receiving BA. As patients were often making radical lifestyle changes and decisions in response to their cardiac event (e.g. stopping smoking, moving home), it was not possible to establish from patients whether they felt CR alone would have had the same effect*.*

Using a range of qualitative methods added depth and breadth to the findings but it was not always possible to make decisions on changes for the pilot study linked to all the findings. Some of the findings were contradictory (e.g. group based versus desire for privacy), and others would not have been practical within the remit of the research funding (e.g. providing all EPC sessions in person and not by phone).

### Practice and evidence

Looking at these findings in the context of other studies, the acceptability of BA seen by practitioners and most patients mirrors previous studies showing that BA is simple and relatively quick to deliver and easy to understand for patients [37]. One recent nested qualitative study aimed to explore an intervention to integrate depression care within patients’ long term condition (LTC) management, using a collaborative care model [22]. In the study, patients reported valuing the feeling of their health being managed more holistically, but both patients and providers expressed a preference for separating the care of mental and physical health issues both in terms of treatment and protected space and time outside of the LTC clinic [22]. In our study, most patients likewise valued the opportunity to discuss their depressive symptoms within the context of their CR. In contrast, most also appreciated the option to receive a mental health treatment and care coordination through their CR nurse, perhaps because of the holistic and restorative nature of the CR programme and the fact that patients were usually still in the more acute stage of their cardiac illness.

A care management model within CR for patients with low mood needed to be versatile enough to allow for the range of physical, mental and social circumstances encountered by many patients seen here. This aspect was also highlighted in a previous qualitative study which explored perceptions of patients with coronary heart disease and depression and their ‘personal and social story of loss’ [38]. Shifting the emphasis onto the care coordination element of EPC in preparation to pilot the intervention aimed to accommodate the issue of possible disruptions in treatment and reduce the need for such intense input from the CR nurse. It also aimed to address the variation in CR pathways that nurses delivered and patients encountered.

## Conclusions

Employing multiple qualitative methods enabled us to revise the intervention in a way that should optimise its acceptability and help nurses to deliver it within a pilot trial by shifting from being a nurse-led to a patient-driven nurse supported model that could be more readily implemented within existing CR services. Despite this shift, the intervention still incorporated both BA and care coordination. In view of the findings, we have reduced emphasis on BA and given mental health care coordination (following NICE guidance) [28] a much greater focus in the pilot study, and thus made a fundamental shift in the profile of the intervention, whilst retaining its core components.

## Additional file

Additional file 1: Topic guides used for participant interviews. (DOCX 27 kb)

## Abbreviations

BACPR: British Association for Cardiovascular Prevention and Rehabilitation
CR: Cardiac rehabilitation
DVD: Digital versatile disk
EPC: Enhanced psychological care
GAD-7: Generalized Anxiety Disorder-7
GP: General practitioner
HADS: Hospital Anxiety and Depression Scale
IAPT: Improving Access to Psychological Therapies
MHCC: Mental health care coordination
NICE: National Institute for Health and Clinical Excellence
PHQ-9: Patient Health Questionnaire-9
PPI: Patient and public involvement
UK: United Kingdom
