# Supplementary material for: Development and refinement of a complex intervention within cardiac rehabilitation services: experiences from the CADENCE feasibility study
Source: Pilot Feasibility Stud. 2017 Feb 13;3:9. doi: 10.1186/s40814-017-0123-1 (PMC5304389; doi:10.1186/s40814-017-0123-1)
Supplement: Additional file 1: — Topic guides used for participant interviews. (DOCX 27 kb) [file 40814_2017_123_MOESM1_ESM.docx]

Additional file 1: Topic guides used for participant interviews

| **Patients** |
| --- |
| - Background and illness history *(About the cardiac event, discharge home – wellbeing physically and mentally after discharge)* |
| - Expectations and decision to come for cardiac rehabilitation *(Decision to attend CR, appropriateness of CR nurse assessing mood and providing support for low mood)* |
| - Experiences of receiving BA sessions and care coordination *(Number of sessions completed, areas covered in BA sessions, areas helpful/ unhelpful, what effect BA had on mood, sessions by telephone rather than face-to-face, physical environment for EPC delivery)* |
| - Using the patient handbook and BA materials *(How the handbook was used, what areas were helpful/could be improved)* |
| - Identifying links between mood and activity level *(Understanding BA, making changes to behaviour)* |
| - Relationship with the CR nurse *(e.g. relaxed, formal etc., perception of nurse’s knowledge/skills to assess mental health and deliver BA treatment)* |
| - Treatment adherence *(Session attendance, what helped to stay engaged)* |
| - How EPC has affected physical and mental well-being *(current mental and physical wellbeing, how best to support mental health, recommending EPC to others and suggestions about more effective EPC delivery)* |
| - Any other treatments received or referrals made during the study and impact on mood |

| **Nurses** |
| --- |
| **Interview 1 (post-training):** |
| - Experiences of the EPC training *(how well tailored to support EPC delivery, teaching style, length)* |
| - Views of the nurse and patient manuals/handbook *(layout, content, how they will use them, any improvements required)* |
| - Implementing Training *(how knowledge gained will help to integrate EPC delivery into current CR programme and workload, any gaps in training, most helpful areas, any changes required in the training)* |
| - Views of EPC (*positive aspects, concerns, anything else needed to support delivery*) |
| **Interview 2 (after delivering the intervention):** |
| - Experiences of delivering BA (*addressing low mood, understanding and explaining BA, areas useful or struggling with; telephone versus face-to-face delivery*) |
| - Using the materials (*e.g. the nurse manual and patient handbook, mood diaries, other tools*) |
| - Training (*Extent to which training was tailored to deliver EPC. Any areas needing more input*) |
| - Managing mental health risk issues and care coordination |
| - Support (*Feedback on supervision sessions*) |
| - Impact on relationship with patients and perceived mental health role |
| - Practicalities (*Findings space, time to deliver EPC, managing nurses’ workloads)* |
| - Impact of integrating EPC on the existing service on wider team(s) (*factors that hinder/help/improve smooth delivery*) |
